# Supplementary material for: Increasing pulse pressure ex vivo, mimicking acute physical exercise, induces smooth muscle cell-mediated de-stiffening of murine aortic segments
Source: Commun Biol. 2023 Nov 9;6:1137. doi: 10.1038/s42003-023-05530-6 (PMC10636049; doi:10.1038/s42003-023-05530-6)

Supplementary Material

Increasing Pulse Pressure *Ex Vivo*, Mimicking Acute Physical Exercise, Induces Smooth Muscle Cell-Mediated De-Stiffening of Murine Aortic Segments

Cédric H. G. Neutel*[1], Anne-Sophie Weyns[2], Arthur Leloup[1], Sofie De Moudt[1], Pieter-Jan Guns[1], Paul Fransen[1]

[1] Laboratory of Physiopharmacology, University of Antwerp, Campus Drie Eiken, Antwerp, Belgium.

[2] Natural Products & Food Research and Analysis – Pharmaceutical technology (NatuRA-PT), University of Antwerp, Campus Drie Eiken, Belgium

*Correspondence: Cédric Neutel, cédric.neutel@uantwerpen.be, Universiteitsplein 1, 2610, Antwerpen, Campus Drie Eiken, University of Antwerp.

Supplementary Figure 1

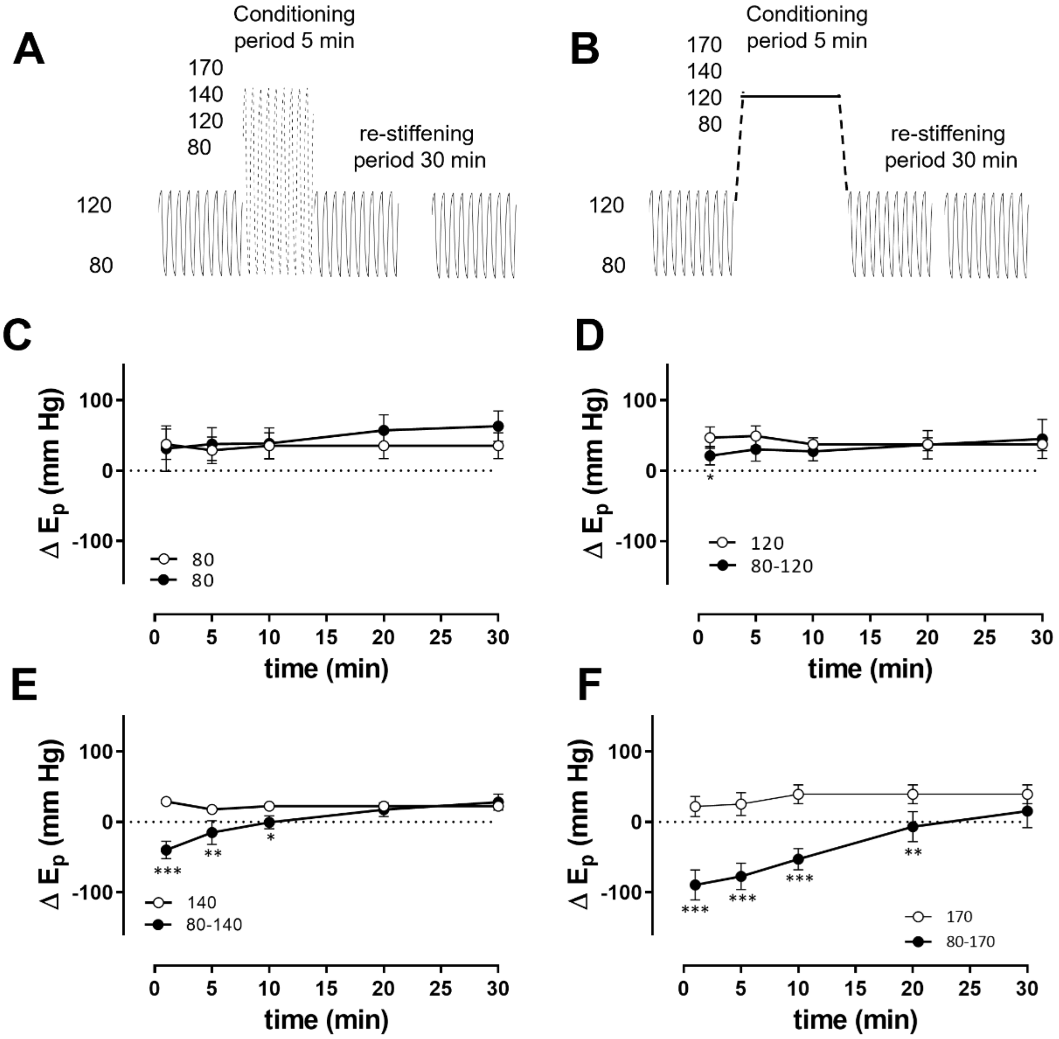

Supplementary figure 1. Static stretch, in contrast to cyclic stretch, does not induce aortic de-stiffening. According to the experimental protocol, aortic segments were challenged with dynamic (A) or static stretch (B) for 10 minutes in the presence of 10 nM PE and 300 µM L-NAME, after which E_p was measured at 80-120 mm Hg (re-stiffening period). Static stretch (open circles) was 80 (C), 120 (D), 140 (E) and 170 (F) mm Hg, dynamic stretch (closed circles) was 0 (80-80, C), 40 (80-120, D), 60 (80-140, E) and 90 (80-170, F) mm Hg. Two-way ANOVA with Sidak's multiple comparison. n=6. *, **, ***: $p<0.05, 0.01, 0.001$ static versus dynamic. **Ep** = Peterson's Modulus of Elasticity

Supplementary Figure 2

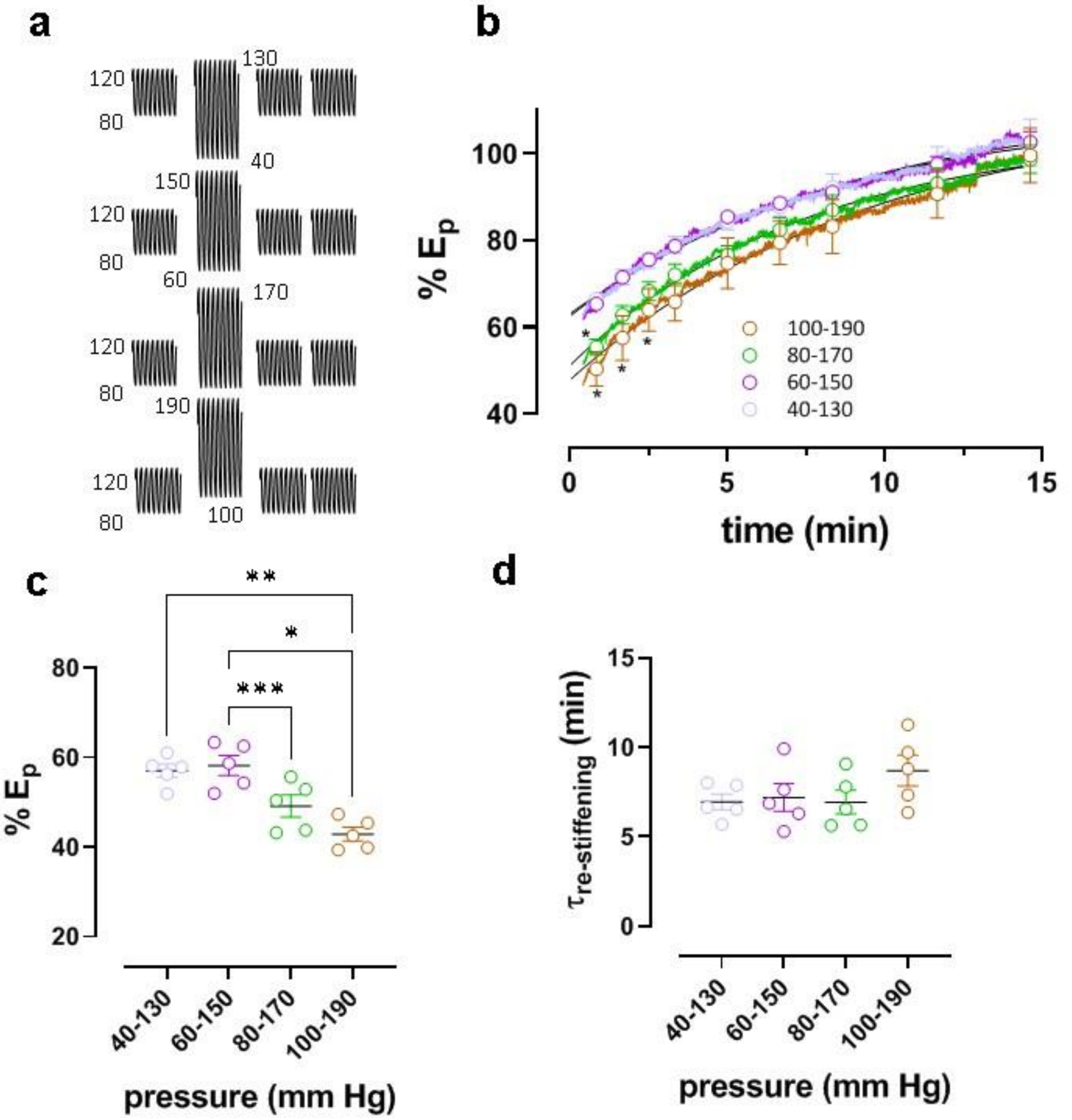

Supplementary figure 2. The interaction between high pulse pressure and high mean pressure in inducing aortic tissue de-stiffening. Aortic segments (n=5) were subjected to pulse pressure of 90 mm Hg at different mean pressures in the presence of 2 µM PE and 300 µM L-NAME (a). After return to 80-120 mm Hg, % E_p was determined with E_p before the high PP of 90 mm Hg as reference. E_p was continuously measured during the post-conditioning period of 20 minutes and fitted with a mono-exponential function (b). Amount of de-stiffening (extrapolated to time = 0 in the mono-exponential increase of E_p) and time constant of the re-stiffening process are shown in c and d. Two way ANOVA with Dunnett's multiple comparison test (b, *: $p<0.05$); one way ANOVA with Tukey's multiple comparison test (c, d: *, **, ***:$p<0.05, 0.01, 0.001$). **Ep** = Peterson's Modulus of Elasticity

3 Supplementary Figure 3

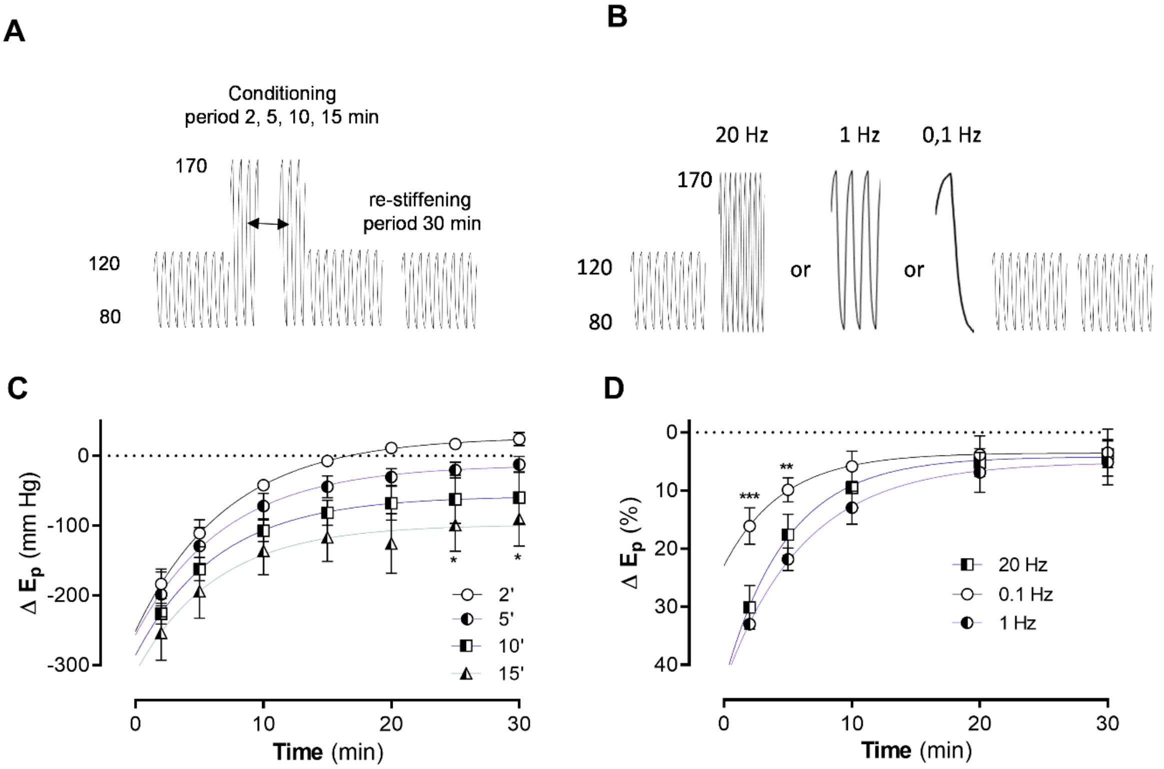

Supplementary figure 3: The effect of conditioning period and pulse frequency on de- and re-stiffening. De-stiffening effects of conditioning periods of 2, 5, 10 and 15 min during which segments (n=4) were stretched at 80-170 mm Hg in the presence of 2 µM PE (A). After conditioning E_p was measured at 2, 5, 10, 15, 20, 25 and 30 minutes with respect to the value before conditioning (ΔEp) (C). *: $p<0.05$, 15 minutes versus 2 minutes, Two-way ANOVA, Tukey's multiple comparison test. De-stiffening effects of conditioning periods of 10 min at 80-170 mm Hg at 0.1, 1 and 20 Hz (n=5) in the presence of 100 nM PE and 300 µM L-NAME (B). After conditioning E_p was measured at 2, 5, 10, 20 and 30 minutes with respect to the value before conditioning (ΔEp) (D). **, ***: $p<0.01$, 0.001 1Hz versus 0.1 Hz. All data points shown are at 80-120 mm Hg. Full lines are mono-exponential fits to the data: $\Delta E_p = \Delta E_{p(0)} + (\Delta E_{p(30)} - \Delta E_{p(0)}) * (1 - \exp(-time/\tau))$ with $\Delta E_{p(0)}$,ΔE_p at time 0 min, $\Delta E_{p(30)}$,ΔE_p at time 30 min and τ, the time constant of re-stiffening. **Ep** = Peterson's Modulus of Elasticity

4 Supplementary Figure 4

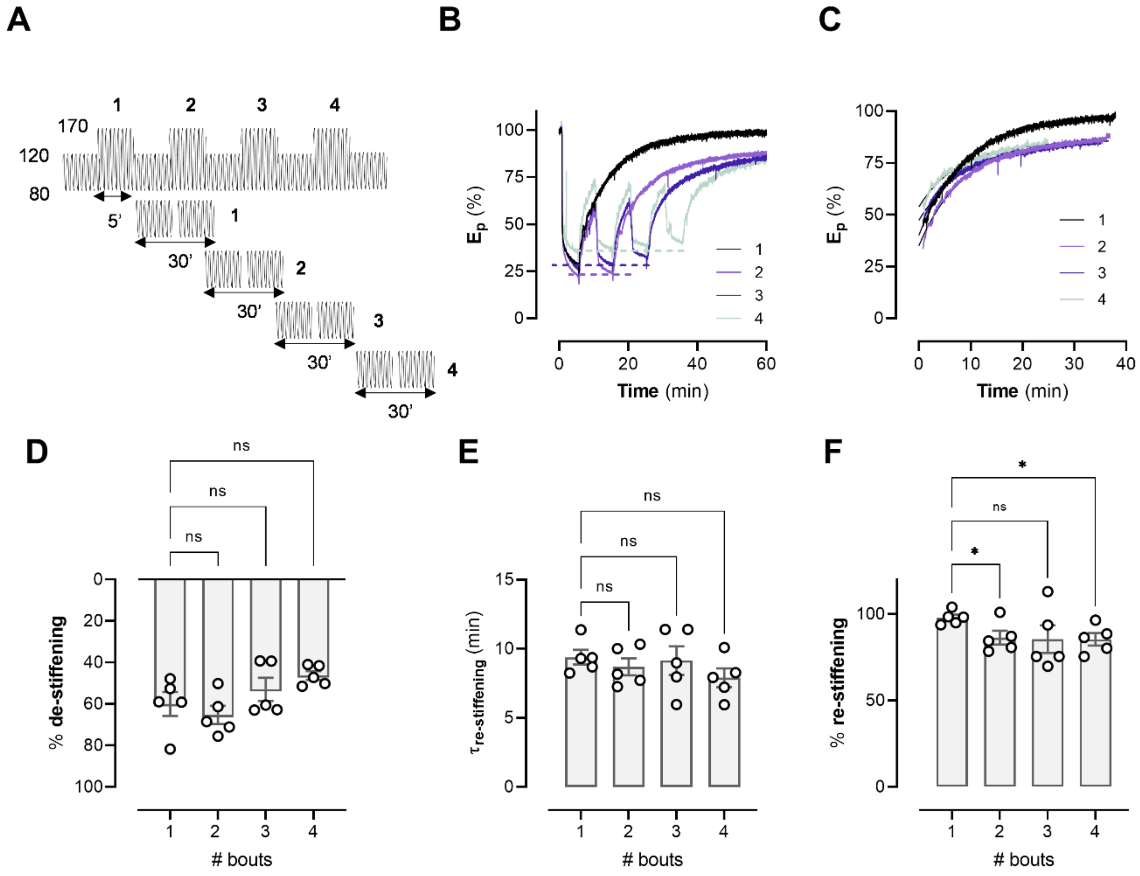

Supplementary figure 4. The number of high pulsatile bouts has effects on the amount of re-stiffening after the conditioning period only. Aortic segments (n=5) were conditioned with 1, 2, 3 or 4 bouts of 90 mm Hg PP between 80 and 170 mm Hg in the presence of 2 µM PE and 300 µM L-NAME. Bouts were separated by "recovery" periods of 5 minutes at normal PP of 40 mm Hg between 80 and 120 mm Hg (A). Absolute and mean relative traces (E_p before conditioning was set to 100%) of 5 aortic segments of 5 mice are shown in (B and C). The mean relative de-stiffening after 1, 2, 3 and 4 bouts, the time constant of re-stiffening and plateau of re-stiffening are shown in D, E and F. One-way ANOVA with Tukey's multiple comparison test (*:p<0.05). **Ep** = Peterson's Modulus of Elasticity

5 Supplementary Figure 5

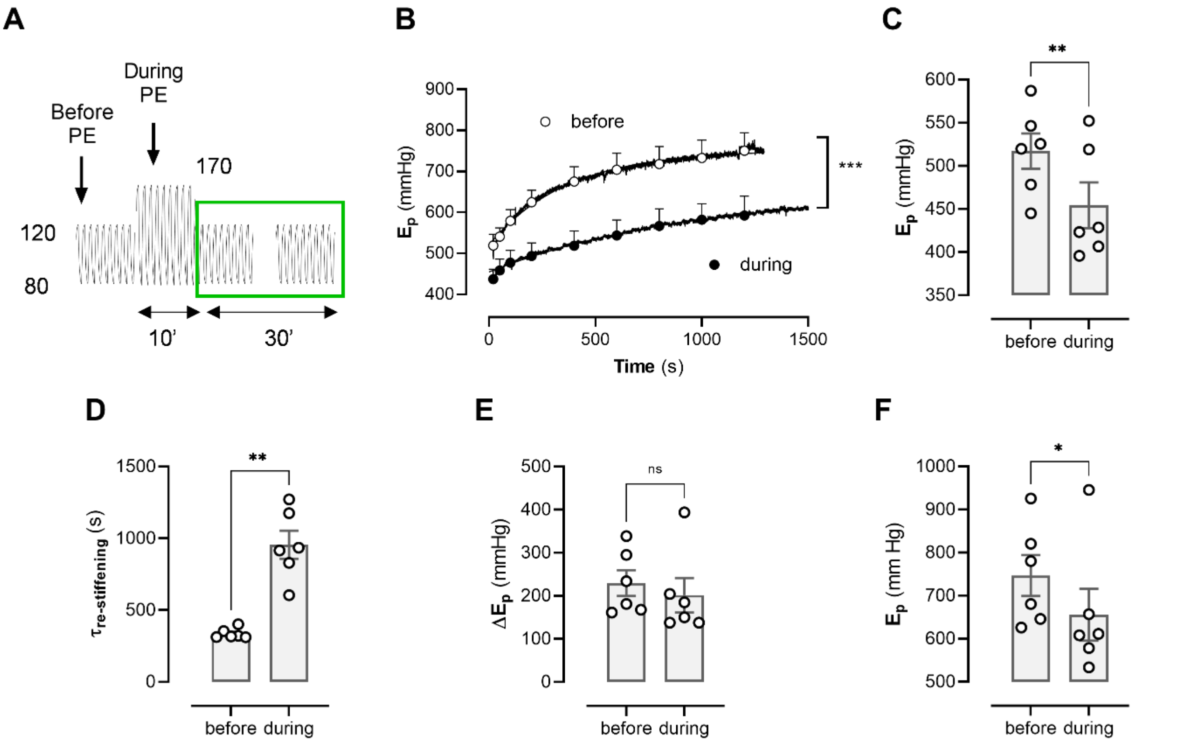

Supplementary figure 5: Time of phenylephrine application affects de- and re-stiffening at 80-120 mm Hg after conditioning at 80-170 mm Hg. 2 µM PE was applied before or during the conditioning of the aortic segments at 80-170 mm Hg for 4 minutes (A). After the conditioning, E_p re-stiffened (B). De-stiffening (C) is significantly larger for "during" than for "before". The time constant (D) is significantly larger for "during" than for "before", indicating slower re-stiffening in the "during" condition. The amount of re-stiffening (E) was not significantly different but E_p re-stiffened to smaller values in the "during" condition (F). Two-way ANOVA with Sidak's multiple comparison test for B, paired t-test for C, D, E and F. n=6. *, **, ***: P<0.05, 0.01, 0.001 during versus before. **PE** = phenylephrine; **Ep** = Peterson's Modulus of Elasticity

6 **Supplementary Figure 6 – Uncropped Blots**

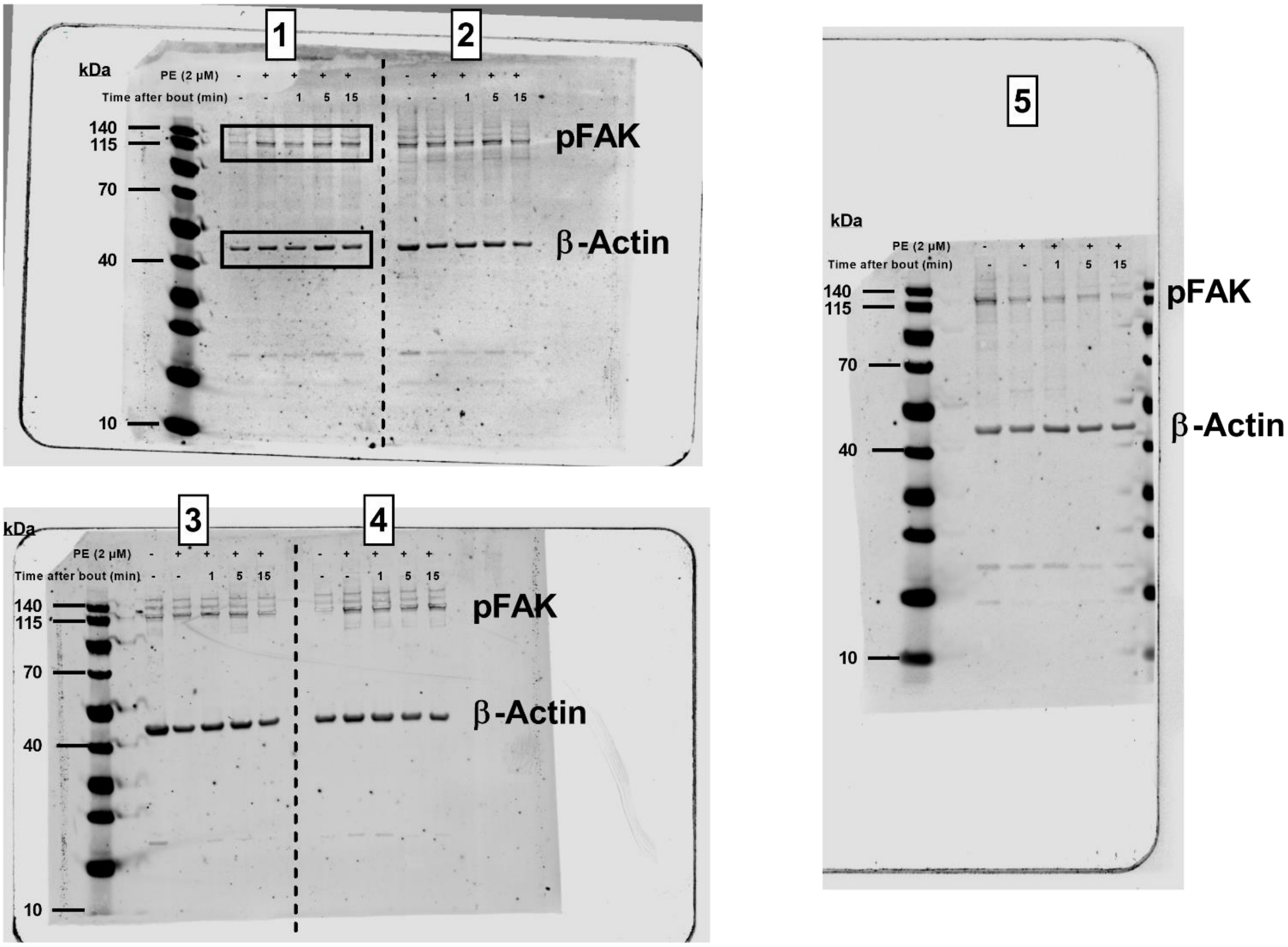

Supplement: Supplementary file 2 — Supplementary Information [file 42003_2023_5530_MOESM2_ESM.pdf]
