# Supplementary material for: Increasing pulse pressure ex vivo, mimicking acute physical exercise, induces smooth muscle cell-mediated de-stiffening of murine aortic segments
Source: Commun Biol. 2023 Nov 9;6:1137. doi: 10.1038/s42003-023-05530-6 (PMC10636049; doi:10.1038/s42003-023-05530-6)
Supplement: Supplementary file 5 — Reporting Summary [file 42003_2023_5530_MOESM5_ESM.pdf]

nature portfolio

Corresponding author(s): Cédric H.G. Neutel

Last updated by author(s): Oct 19, 2023

Reporting Summary

Nature Portfolio wishes to improve the reproducibility of the work that we publish. This form provides structure for consistency and transparency in reporting. For further information on Nature Portfolio policies, see our Editorial Policies and the Editorial Policy Checklist.

Statistics

For all statistical analyses, confirm that the following items are present in the figure legend, table legend, main text, or Methods section.

n/a	Confirmed	
☐	☒	The exact sample size (*n*) for each experimental group/condition, given as a discrete number and unit of measurement
☐	☒	A statement on whether measurements were taken from distinct samples or whether the same sample was measured repeatedly
☐	☒	The statistical test(s) used AND whether they are one- or two-sided<br>*Only common tests should be described solely by name; describe more complex techniques in the Methods section.*
☐	☒	A description of all covariates tested
☐	☒	A description of any assumptions or corrections, such as tests of normality and adjustment for multiple comparisons
☐	☒	A full description of the statistical parameters including central tendency (e.g. means) or other basic estimates (e.g. regression coefficient) AND variation (e.g. standard deviation) or associated estimates of uncertainty (e.g. confidence intervals)
☒	☐	For null hypothesis testing, the test statistic (e.g. *F*, *t*, *r*) with confidence intervals, effect sizes, degrees of freedom and *P* value noted<br>*Give P values as exact values whenever suitable.*
☒	☐	For Bayesian analysis, information on the choice of priors and Markov chain Monte Carlo settings
☒	☐	For hierarchical and complex designs, identification of the appropriate level for tests and full reporting of outcomes
☒	☐	Estimates of effect sizes (e.g. Cohen's *d*, Pearson's *r*), indicating how they were calculated

Our web collection on statistics for biologists contains articles on many of the points above.

Software and code

Policy information about availability of computer code

Data collection	Labchart 8, Universal Grap 6.1
Data analysis	GraphPad Prism 8

For manuscripts utilizing custom algorithms or software that are central to the research but not yet described in published literature, software must be made available to editors and reviewers. We strongly encourage code deposition in a community repository (e.g. GitHub). See the Nature Portfolio guidelines for submitting code & software for further information.

Data

Policy information about availability of data

All manuscripts must include a data availability statement. This statement should provide the following information, where applicable:

- Accession codes, unique identifiers, or web links for publicly available datasets
- A description of any restrictions on data availability
- For clinical datasets or third party data, please ensure that the statement adheres to our policy

The uncropped/unedited western blot images are included in Supplementary Fig. 6. The datasets generated during and/or analyzed during the current study are available from the corresponding author on reasonable request.

Research involving human participants, their data, or biological material

Policy information about studies with human participants or human data. See also policy information about sex, gender (identity/presentation), and sexual orientation and race, ethnicity and racism.

Reporting on sex and gender	This information has not been collected
Reporting on race, ethnicity, or other socially relevant groupings	This information has not been collected
Population characteristics	This information has not been collected
Recruitment	This information has not been collected
Ethics oversight	This information has not been collected

Note that full information on the approval of the study protocol must also be provided in the manuscript.

Field-specific reporting

Please select the one below that is the best fit for your research. If you are not sure, read the appropriate sections before making your selection.

☒ Life sciences ☐ Behavioural & social sciences ☐ Ecological, evolutionary & environmental sciences

For a reference copy of the document with all sections, see nature.com/documents/nr-reporting-summary-flat.pdf

Life sciences study design

All studies must disclose on these points even when the disclosure is negative.

Sample size	Sample size was determined using a statistical power calculator (G*Power)
Data exclusions	all data were included in the analysis, unless the data was influenced by experimental error and was identified as outlier during statistical testing in Graphpad Prism software
Replication	All attempts at replication were succesful
Randomization	Randomization was not relevant to the study as no study treatments needed to be randomized
Blinding	Blinding was not relevant to the study as the main output parameters were not subject to observer bias

Reporting for specific materials, systems and methods

We require information from authors about some types of materials, experimental systems and methods used in many studies. Here, indicate whether each material, system or method listed is relevant to your study. If you are not sure if a list item applies to your research, read the appropriate section before selecting a response.

Materials & experimental systems

n/a	Involved in the study	
☐	☒	Antibodies
☒	☐	Eukaryotic cell lines
☒	☐	Palaeontology and archaeology
☐	☒	Animals and other organisms
☒	☐	Clinical data
☒	☐	Dual use research of concern
☒	☐	Plants

Methods

n/a	Involved in the study	
☒	☐	ChIP-seq
☒	☐	Flow cytometry
☒	☐	MRI-based neuroimaging

Antibodies

Antibodies used	anti-FAK (phosphorylated Tyr397) (Abcam, ab81298), anti- beta-actin (Abcam, ab8226), (IR)-conjugated secondary antibodies (anti-rabbit: IgG926-32211 and anti-mouse: IgG926-68070; Li-Cor Biosciences)

Validation	No information on the validation of the primary antibodies is available

Animals and other research organisms

Policy information about studies involving animals; ARRIVE guidelines recommended for reporting animal research, and Sex and Gender in Research

Laboratory animals	C57Bl6/J mice were used, n=58, 32 female, 26 male, age between 4 and 7 months
Wild animals	n/a
Reporting on sex	Sex differences were not systematically looked for in the current study. While there might be differences between both sexes in the magnitude of the responses, our data in males and females never differed in the way the aortic segment responded to the different experimental protocols.
Field-collected samples	n/a
Ethics oversight	n/a

Note that full information on the approval of the study protocol must also be provided in the manuscript.

Plants

Seed stocks	n/a
Novel plant genotypes	n/a
Authentication	n/a
